# Effects of different shaping processes on the quality, volatile and non-volatile metabolites of Xinyang Maojian green tea

**DOI:** 10.1016/j.fochx.2026.104722

**Published:** 2026-10-28

**Authors:** Jinlei Luo, Yi Chen, Lingzhi Chen, Peng Yin, Guiyi Guo, Zhihui Wang

**Affiliations:** a School of Tea Science, Xinyang Agriculture and Forestry University, Key Laboratory of Development and Utilization of Tea and Characteristic Plant Resources in South Henan, Xinyang, Henan, China; b Jiangxi Cash Crops Research Institute, Nanchang, Jiangxi, China

**Keywords:** Xinyang Maojian green tea, Tea shaping processes, Metabolomics, Electronic nose and tongue, Sensory quality

## Abstract

Shaping processes critically influence green tea sensory quality. This study examined four shaping methods – No Shaping (NS), Chao Tiao (CT), Pai Ba (PB), and Li Tiao (LT) - on Xinyang Maojian green tea using sensory evaluation, physicochemical assays, GC–MS, and LC-MS. CT induced the most profound reconfiguration: it enhanced chestnut/beany aromas and umami while reducing bitterness/astringency. CT had the highest water extract and free amino acids, with ester-type catechins elevated by approximately 15% relative to NS. GC–MS showed CT up-regulated heterocyclic compounds (e.g., pyrazoles) and down-regulated terpenoids. LC-MS revealed CT elevated umami-enhancing di/tri-peptides and reduced astringent phenolic compounds (e.g., the phenolic acid caffeic acid). LT broadly accumulated small peptides/phenolic glycosides, supporting fuller mouthfeel and sweet aftertaste. PB and NS showed limited changes. These results demonstrate CT as a precise regulator linking physical disruption to metabolic reprogramming for desirable chestnut/beany characteristics, providing a basis for targeted green tea quality improvement.

## 1 Introduction

Green tea is one of the most widely consumed beverages globally, prized for its unique flavor, aroma, and numerous health benefits (Zhai et al., 2022). The quality of green tea is profoundly influenced by the processing steps, particularly the fixation (shaqing), rolling, shaping, and drying stages (Singh et al., 2014; Xu et al., 2019). Among these, the shaping process is critical as it determines the final appearance, texture, and internal chemical composition of the tea leaves, which directly impacts the sensory characteristics of the infusion (Qiu et al., 2021).

Xinyang Maojian (XYMJ) is a famous Chinese green tea, renowned for its unique “tight, thin, round, and straight” shape and its strong, chestnut-like aroma and mellow taste (Cui et al., 2022; Wang et al., 2021a). In the traditional production of XYMJ, the shaping process is a labor-intensive and skill-dependent step. Modern mechanization has introduced several shaping methods to standardize production and improve efficiency. The most common mechanical shaping methods for XYMJ include Chao Tiao (CT), which uses a rolling-and-stir-frying machine that mimics manual shaping; Pai Ba (PB), which applies gentler stirring and kneading to shape the leaves; and Li Tiao (LT), which uses reciprocating vibration to straighten the leaves into thread-like strips (Yin et al., 2022).

Given that mechanical forces during processing (e.g., compression, shear, and thermal input) are known to disrupt cellular structures and accelerate enzymatic/non-enzymatic transformations of metabolites such as catechins, amino acids, and volatile compounds (Wen et al., 2022; Zeng et al., 2019), shaping processes, which involve distinct mechanical actions, may significantly influence the final quality of green tea. Over the past few years, the specific influence of shaping techniques on green tea quality has received growing interest. Zhou et al. (2025) used a multi-platform approach integrating electronic tongue, colorimetric analysis, and untargeted metabolomics to compare different shaping techniques, and demonstrated that shaping practices substantially remodel the non-volatile metabolite profiles and sensory quality of green tea. Tang et al. (2025) employed GC-*E*-Nose and GC–MS/MS to reveal that different shaping methods distinctly regulate volatile metabolites and aroma quality. Nevertheless, these studies were mostly conducted on generic green tea products and focused on either non-volatile or volatile components separately, without an integrated assessment combining sensory, physicochemical, and both volatile and non-volatile dimensions. Multi-platform analytical approaches, including electronic tongue (*E*-tongue), electronic nose (E-nose), gas chromatography–mass spectrometry (GC–MS), and liquid chromatography-mass spectrometry (LC-MS), have been successfully applied to characterize the flavor and chemical complexity of tea (Li et al., 2022; Peng et al., 2023), yet such a comprehensive strategy has not been systematically employed to dissect the effects of shaping techniques on tea quality. In addition, previous studies on XYMJ have mainly focused on rolling or drying parameters (Li et al., 2024), and the impact of its locally used shaping methods (CT, PB, and LT) on the comprehensive quality has remained unexplored.

To address this knowledge gap, we conducted a systematic study comparing XYMJ green tea samples processed with no shaping (NS, control), CT, PB, and LT. By integrating sensory evaluation, physicochemical indicators, volatile profiling, and non-volatile metabolomics, we aimed to identify the key compounds responsible for aroma and taste differences among shaping methods and to provide a scientific basis for optimizing industrial processing of premium XYMJ green tea.

## 2 Material and methods

### 2.1 Processing

The green tea samples with different shaping processes were prepared and obtained according to the scheme shown in Fig. 1. Fresh tea shoots (one bud with one leaf) were harvested from local tea plantations in Shihegang, Xinyang City (Henan Province, China), spread evenly on bamboo trays for a spreading process of 4 h, and then subjected to fixation using a drum fixation machine (Model: 6CT-60, Xinyang Yongheng Tea Technology Co., Ltd., China) at 200 °C for 2 min. After cooling to room temperature, the fixed leaves were rolled in a rolling machine (Model: 6CR-40, Xinyang Yongheng Tea Technology Co., Ltd., China) for 20 min using a “light-heavy-light” pressure pattern. The rolled leaves were then divided into four groups and subjected to different shaping treatments: Chao Tiao (CT), Pai Ba (PB), and Li Tiao (LT) were each applied for 5 min using three different shaping machines, whereas the no-shaping control (NS) received no shaping and was transferred directly to the drying step. Specifically, CT was shaped on a Chao Tiao machine (Model: 6CCG72, Xinyang Huayang Industry and Trade (Leifeng Tea Machinery Factory), China) at a rotation speed of approximately 140 rpm. PB was shaped on a Pai Ba machine (Model: 6CCGK-60, Xinyang Yiding Tea Technology Co., Ltd., China) at a rotation speed of approximately 70 rpm. LT was shaped on a Li Tiao machine (Model: 6CCB-80, Xinyang Yongheng Tea Technology Co., Ltd., China) at a reciprocating frequency of approximately 140 times/min. During the shaping stage, all three shaped treatments were carried out at a measured leaf temperature of approximately 65 °C, which was maintained by adjusting the machine-set temperature accordingly (monitored with a BS400 infrared thermometer, BS, USA). The thermal conditions during shaping were therefore comparable among CT, PB, and LT, and the observed differences among these treatments can be largely attributed to the distinct modes of mechanical action. The shaping machines were operated at their standard industrial parameters commonly used in local Xinyang Maojian production. Since they were not equipped with pressure sensors, the applied pressure was not recorded. These parameters are representative of commercial practice. After shaping (or direct transfer for NS), all samples were dried using a drying machine (Model: CS-6HZ-6, Xinyang Yongheng Tea Technology Co., Ltd., China) in a two-stage process: initial drying at 70 °C for 30 min, followed by final drying at 70 °C for an additional 20 min. The finished dried green tea samples were immediately sealed and stored at −20 °C until subsequent analysis.

**Fig. 1:**
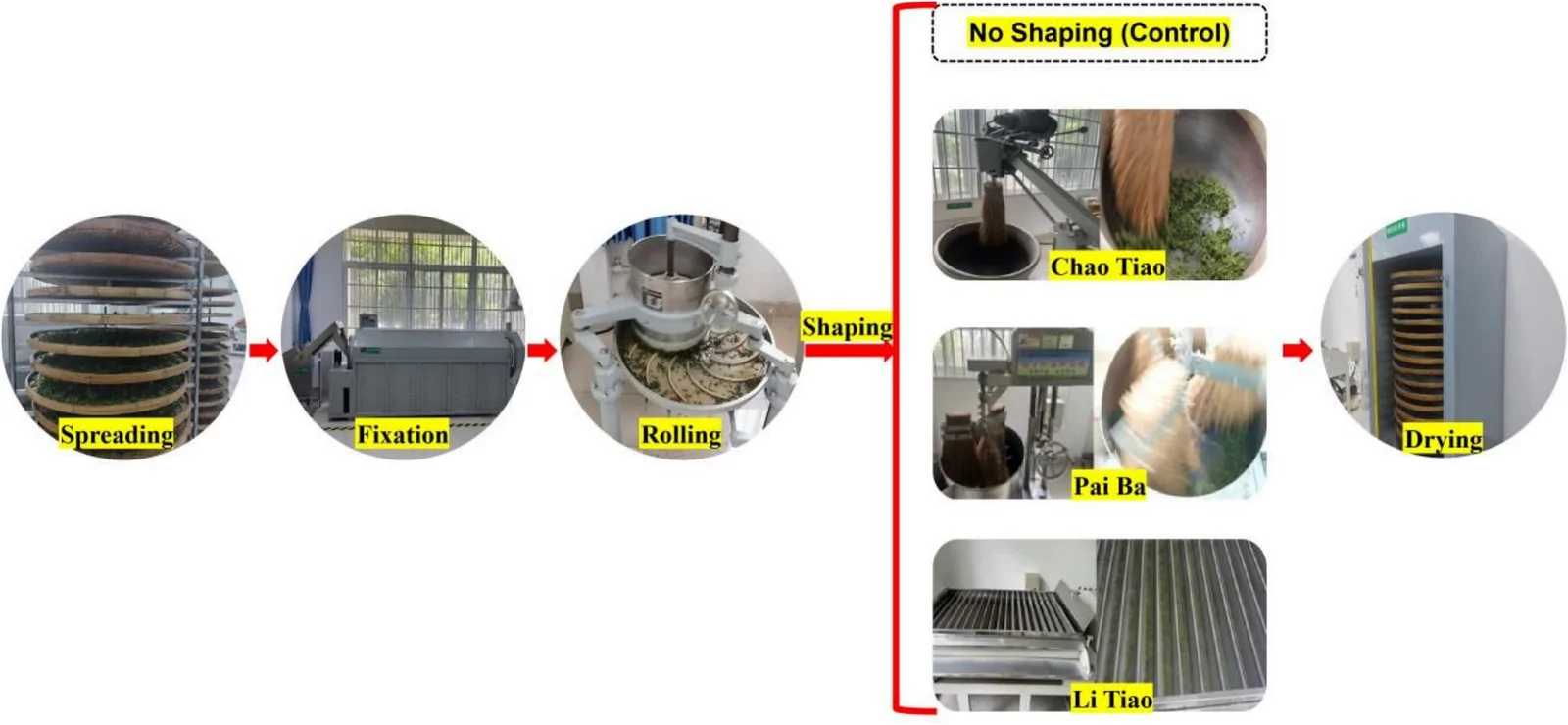
Schematic diagram of the processing technology for green tea samples under different shaping treatments. (For interpretation of the references to color in this figure legend, the reader is referred to the web version of this article.)

### 2.2 Sensory evaluation

Quantitative descriptive analysis (QDA) was conducted by a panel of 12 well-trained assessors (6 males and 6 females, aged 20–55 years) from the Tea Quality Research Group of Xinyang Agriculture and Forestry University, College of Tea Science, following the method described by Wang et al. (2024) with modifications. Briefly, 3.0 g of uniformly mixed dry tea sample from each treatment was placed into a 150 mL cylindrical evaluation cup. Then, 150 mL of freshly boiled distilled water was added, and the sample was steeped for 5 min. After steeping, the tea infusion was decanted into a tasting bowl for evaluation by each panelist. Ten descriptors with a usage rate exceeding 80% were selected from preliminary discussions, including five aroma attributes (grassy, fresh, floral, chestnut, beany) and five taste attributes (bitterness, astringency, umami, thickness, sweet aftertaste). The tea infusion was re-prepared following the same method for each replicate. Panelists evaluated the intensity of each attribute using a 0–7 point scale, where 0 = none, 4 = moderate, and 7 = extremely strong. The intensity score for each attribute and the overall taste acceptance of each sample were calculated as the mean value of all panelists' ratings. Each dry tea sample was assessed in three independent biological replicates.

### 2.3 Determination of electronic tongue and electronic nose

The tea infusion was prepared following the same procedure as sensory evaluation and maintained at 50 °C. The electronic tongue (Bosin Technology, Shanghai, China) was equipped with three working electrodes: S1 (gold), S2 (platinum), and S3 (silver), along with an Ag/AgCl reference electrode and a platinum auxiliary electrode. Before measurement, the sensor was immersed in ultrapure water for 10 min and then placed in a standard reference solution to stabilize the baseline. Two pre-experiments were conducted to adjust the sensor parameters, ensuring that the maximum signal fell within ±0.1–2.5. For each sample, the sensor was cleaned with ultrapure water for 120 s, followed by a 30 s measurement period. The signals of basic taste standard solutions were recorded, and drift compensation was applied using the instrument software. The electronic nose (cNose-18, Bosheng Technology, Shanghai, China) contained a 10-sensor metal-oxide semiconductor array responsive to nitrogen oxides, sulfides, alcohols, aldehydes, terpenes, and aromatic compounds. Approximately 3 g of tea leaves were placed into a 50 mL sealed vial and equilibrated at 50 °C for 10 min. The headspace gas was then pumped into the sensor chamber at 400 mL/min for 120 s, and the stable segment (last 20 s) of the conductance ratio (G/G₀) was used for analysis. For both instruments, three separate infusions were prepared for each treatment, and each infusion was measured three times. The average values of the replicate measurements were used for subsequent data analysis.

### 2.4 Physicochemical analysis

The water extract content was determined by hot water extraction. Briefly, 2.000 g of ground tea sample was extracted with 300 mL of boiling distilled water in a boiling water bath for 45 min, with occasional shaking every 10 min. After vacuum filtration, the residue was washed with boiling water, dried at 120 ± 2 °C for 1 h twice, and weighed. The water extract content was calculated as the difference between the initial dry tea weight and the final residue weight, expressed as a percentage of the dry weight. The total free amino acid content was analyzed using the ninhydrin colorimetric method, with the absorbance measured at 570 nm, using glutamic acid as the standard. The total polyphenol content was measured by the Folin-Ciocalteu colorimetric method at 760 nm, with gallic acid as the standard. Soluble sugar content was determined by the anthrone‑sulfuric acid colorimetric method at 620 nm, using glucose as the standard. The total flavonoid content was quantified by the aluminum trichloride colorimetric method, with absorbance recorded at 510 nm, using rutin as the standard. Caffeine and individual catechins (including epigallocatechin gallate (EGCG), gallocatechin gallate (GCG), epicatechin gallate (ECG), epigallocatechin (EGC), epicatechin (EC), and catechin (C)) were simultaneously determined by high-performance liquid chromatography (HPLC) as described by Deng et al. (2024).

### 2.5 Analysis of the volatiles present in tea samples

#### 2.5.1 Extraction of volatiles using HS-SPME

Tea samples (500 mg) were accurately weighed into a 20 mL headspace vial, after which 2 mL of saturated sodium chloride solution and 20 μL of internal standard solution (10 μg/mL) were added and the vial was immediately sealed. For HS-SPME, the vial was equilibrated in an agitator at 60 °C for 5 min, and a pre-aged SPME Arrow (120 μm DVB/CWR/PDMS, Agilent, USA) was then inserted into the headspace for extraction at 60 °C for 15 min. Before first use, the fiber was conditioned at 250 °C for 2 h and re-conditioned at 250 °C for 5 min before each subsequent analysis. After extraction, the fiber was immediately desorbed in the GC injection port at 250 °C for 5 min in splitless mode.

#### 2.5.2 GC–MS analysis

Volatile analysis was performed using an Agilent 8890 GC system coupled to an Agilent 7000D triple-quadrupole mass spectrometer (Agilent Technologies, USA), which was operated in single-quadrupole (GC–MS) mode. Separation was achieved on a DB-5MS capillary column (30 m × 0.25 mm × 0.25 μm, Agilent, USA) with high-purity helium (≥99.999%) as the carrier gas at a constant flow rate of 1.2 mL/min. The injector temperature was 250 °C, and splitless injection was used with a solvent delay of 3.5 min. The oven temperature was programmed as follows: 40 °C held for 3.5 min, raised to 100 °C at 10 °C/min, then to 180 °C at 7 °C/min, and finally to 280 °C at 25 °C/min, held for 5 min. The mass spectrometer was operated in electron impact (EI) mode at 70 eV, with the ion source, quadrupole, and transfer line temperatures set at 230 °C, 150 °C, and 280 °C, respectively. For wide-target volatile metabolome profiling, data acquisition was performed in selected ion monitoring (SIM) mode using a time-segmented acquisition strategy optimized by integrating the SIM-based method described in the Chinese national standard GB 23200.8–2016 with the widely-targeted volatilomics approach established by Yuan et al. (2022).

#### 2.5.3 Qualitative and quantitative analyses

An in-house volatile metabolite database was constructed by analyzing authentic standards and representative tea samples in full-scan mode. Compounds were identified by matching the acquired mass spectra against the NIST 20 library with a minimum match factor of 70. Retention indices (RIs) were calculated from the retention times of a C7–C40 n-alkane series using the Van Den Dool–Kratz equation and compared with literature values (acceptable deviation: ±30 RI units). Authentic standards were also used for identification where available. In the sample-screening stage, one quantifier ion and two to three qualifier ions were monitored per compound in SIM mode. Because SIM records only preselected characteristic ions rather than complete mass spectra, NIST match scores were not applicable to the SIM screening data. A compound was therefore considered positively identified when its retention time was consistent with the in-house database value and the quantifier and qualifier ions were simultaneously present. The content of each volatile compound (Xi, μg/g) was calculated using the internal standard semi-quantitative method as follows: X_i_ = (V_s_ × C_s_ × I_i_) / (M × I_s_) × 10^−3^, where X_i_ represents the content of any compound (μg/g), V_s_ is the volume of internal standard solution (L), C_s_ is the concentration of internal standard (μg/mL), M is the sample weight (g), I_i_ and I_s_ are the peak areas of the target compound and internal standard, respectively.

#### 2.5.4 Data preprocessing and statistical analysis

Missing values in the raw data matrix were imputed with 1/5 of the minimum value for each metabolite. Quality control (QC) samples were prepared by pooling equal aliquots of all test samples and inserted every 10 injections to monitor system stability. Metabolites with a coefficient of variation (CV) > 0.5 in QC samples were excluded from further analysis. Partial least squares discriminant analysis (PLS-DA) was performed on the Metware Cloud platform (https://cloud.metware.cn). Differentially abundant volatile metabolites were identified based on variable importance in projection (VIP) scores and *p*-values (VIP > 1, *p* < 0.05). It should also be noted that untargeted GC–MS metabolomics is inherently associated with a risk of metabolite misidentification, especially for structural isomers and compounds with insufficient mass spectral library match information. Although multiple annotation filters were applied to minimize this risk, the reported annotations should be regarded as putative rather than definitive. Definitive identification requires confirmation with authentic reference standards or targeted GC–MS/MS analysis.

### 2.6 Analysis of the non-volatiles present in tea samples

#### 2.6.1 Sample preparation and extraction

Tea samples were vacuum freeze-dried in a Scientz-100F freeze dryer (Ningbo Scientz Biotechnology Co., Ltd., China) for 63 h. The freeze-dried leaves were crushed into fine powder using a ball mill (MM 400, Retsch, Germany) at 30 Hz for 1.5 min. The resulting powder (50 mg) was accurately weighed and mixed with 1200 μL of pre-cooled (−20 °C) 70% aqueous methanol extraction solution containing internal standard. The mixture was vortexed for 30 s every 30 min, with six cycles in total. Following centrifugation at 12,000 rpm for 3 min, the supernatant was collected and filtered through a 0.22 μm microporous membrane prior to UPLC-MS/MS analysis.

#### 2.6.2 UPLC conditions

Sample extracts were analyzed using an ultra-high performance liquid chromatography system (LC-30 A, Shimadzu, Japan) coupled with a quadrupole time-of-flight mass spectrometer (TripleTOF 6600+, SCIEX, USA). Separation was performed on a Waters ACQUITY Premier HSS T3 column (1.8 μm, 2.1 mm × 100 mm). The mobile phase consisted of 0.1% formic acid in water (Phase A) and 0.1% formic acid in acetonitrile (Phase B). The column oven temperature was set to 40 °C, the flow rate was 0.4 mL/min, and the injection volume was 4 μL. The linear gradient elution program was as follows: 0–2.0 min, 5% → 20% B. 2.0–5.0 min, 20% → 60% B. 5.0–6.0 min, 60% → 99% B. 6.0–7.5 min, maintained at 99% B. 7.5–7.6 min, 99% → 5% B. 7.6–10.0 min, maintained at 5% B.

#### 2.6.3 ESI-QTOF-MS/MS conditions

Metabolite detection was carried out in both positive (ESI^+^) and negative (ESI^−^) electrospray ionization modes with an acquisition time of 10 min for each mode. The mass spectrometry parameters were as follows: ion spray voltage, 5000 V (ESI^+^) and − 4000 V (ESI^−^). ion source temperature, 550 °C (ESI^+^) and 450 °C (ESI^−^). nebulizer gas (Gas 1), 50 psi. Auxiliary gas (Gas 2), 60 psi. Curtain gas, 35 psi. Declustering potential (DP), 60 V (ESI^+^) and − 60 V (ESI^−^). MS^1^ collision energy, 10 V (ESI^+^) and − 10 V (ESI^−^). MS^2^ collision energy, 30 V (ESI^+^) and − 30 V (ESI^−^). collision energy spread, 15 V.

#### 2.6.4 Data preprocessing, metabolite identification, and statistical analysis

The original mass spectral data were converted to mzXML format using ProteoWizard software. Peak extraction, alignment, and retention time correction were performed via the XCMS program. Peaks with a missing rate higher than 50% were removed, and blank values were imputed using the K-nearest neighbor (KNN) algorithm. The signal intensities of characteristic peaks were corrected by the support vector regression (SVR) method. Metabolite annotation was performed by searching against in-house, public, and prediction libraries combined with the metDNA method. Metabolites with a comprehensive matching score > 0.5 and a coefficient of variation (CV) < 0.5 in quality control (QC) samples were retained. Finally, datasets from ESI^+^ and ESI^−^ modes were merged, and the metabolite with the highest annotation confidence and the lowest CV value was selected for subsequent analysis. It should also be noted that untargeted LC-MS metabolomics carries inherent risks of metabolite misidentification, especially for structural isomers, co-eluting compounds, and features with insufficient MS/MS library match information. Although multiple annotation filters were applied, the reported non-volatile annotations should be regarded as putative rather than definitive unless confirmed with authentic reference standards or targeted LC-MS/MS analysis. Partial least squares discriminant analysis (PLS-DA) and hierarchical clustering analysis (HCA) were performed on the Metware Cloud platform (https://cloud.metware.cn). Differentially abundant metabolites were identified based on VIP scores and *p*-values. HCA was applied to the differential non-volatile metabolites (VIP > 1.5, *p* < 0.05) using row-wise *Z*-score normalized mean relative abundances, with Euclidean distance and complete linkage clustering. This stricter VIP > 1.5 threshold, compared with VIP > 1 for the volatile metabolites, was used because the LC-MS non-volatile dataset contained a much larger number of annotated features and thus required more stringent filtering to reduce false positives and retain robust discriminants. Given the use of different missing-value strategies for the volatile and non-volatile datasets, the robustness of the overall conclusions to imputation was assessed by comparing PCA score plots obtained for both datasets using the current and alternative imputation strategies. The separation patterns were essentially unchanged (Fig. S1), indicating that the imputation strategy did not materially affect the overall interpretation.

### 2.7 Statistical analysis

All experiments and sample analyses were performed in three independent biological replicates, and the results were expressed as the mean ± standard deviation (SD). One-way analysis of variance (ANOVA) was conducted using SPSS software to evaluate significant differences among sample groups. When ANOVA indicated a significant effect (*p* < 0.05), Duncan's new multiple range test was applied for post-hoc pairwise comparisons. A *p*-value of less than 0.05 was considered statistically significant. Pearson correlation analysis was performed in R (version 4.2.1) to examine the relationships between *E*-nose sensor responses and aroma attributes and between E-tongue sensor responses and taste attributes. Correlation coefficients and *p*-values were visualized as heatmaps using ggplot2 (version 3.4.4).

## 3 Results and discussion

### 3.1 Sensory evaluation of Xinyang maojian green tea under different shaping processes

The four shaping methods produced distinct visual characteristics (Fig. 2A). The No Shaping (NS) sample exhibited loose, curly leaves with a duller green color, while the mechanically shaped teas displayed more uniform and compact forms. Specifically, the Chao Tiao (CT) sample was tightly twisted with a slightly curved shape, the Pai Ba (PB) sample appeared as flat, straight strips, and the Li Tiao (LT) sample formed fine, needle-like strands. All infusions were clear and bright yellow-green, but the liquor color depth varied noticeably: the CT infusion was the deepest, followed by LT (slightly deeper than PB) and PB, with the NS infusion being the lightest. This deepening of liquor color in mechanically shaped samples, especially CT, likely results from enhanced cell disruption and subsequent limited enzymatic oxidation of polyphenols during intense mechanical action (Li et al., 2023). The infused leaves from mechanically shaped samples expanded more evenly and remained more intact compared to the NS control.

**Fig. 2:**
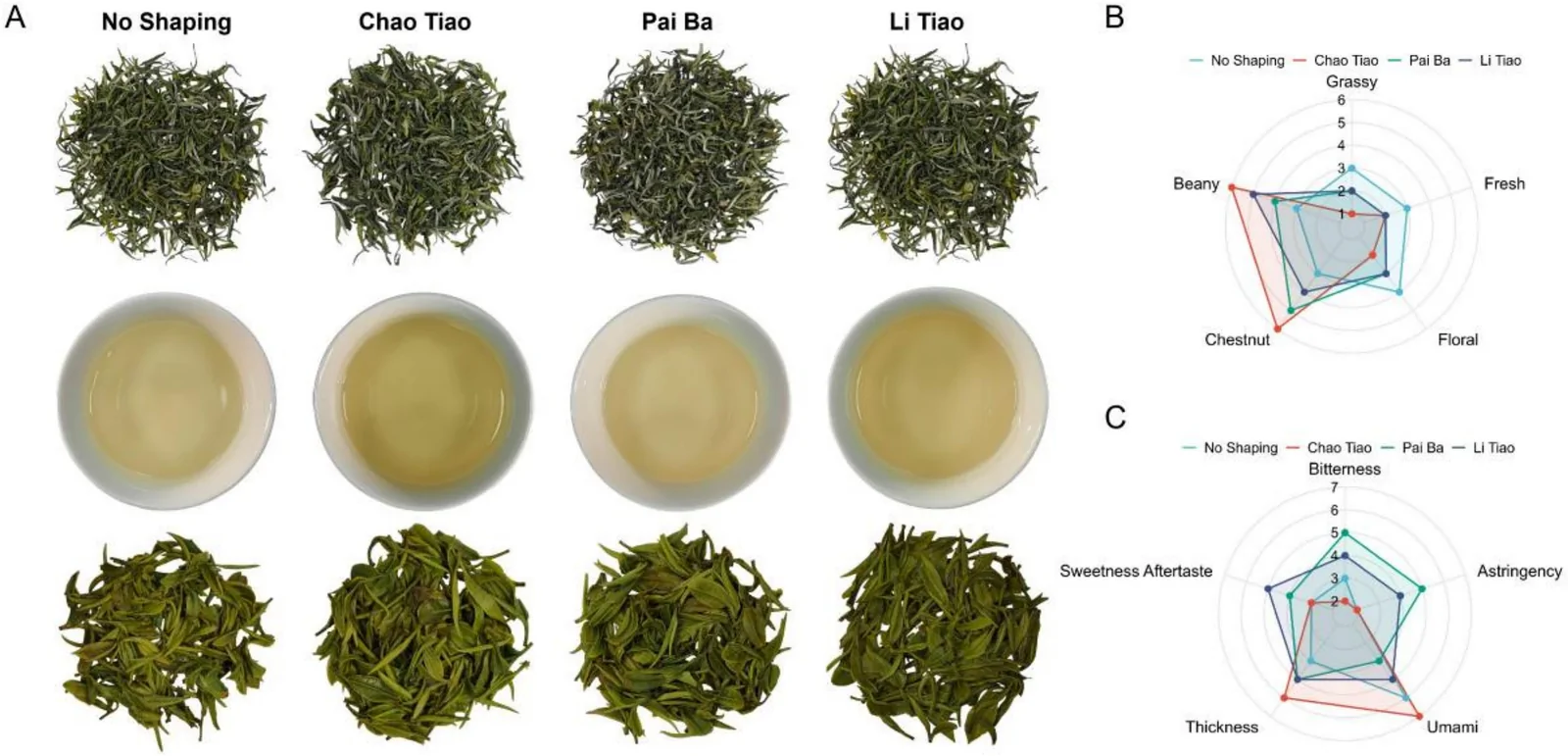
Sensory evaluation results of Xinyang Maojian green tea processed with different shaping methods. (A) Photographs of dry tea leaves, infused leaves, and tea infusions. (B) Radar chart of aroma attributes (grassy, fresh, floral, chestnut, beany). (C) Radar chart of taste attributes (bitterness, astringency, umami, thickness, sweet aftertaste). (For interpretation of the references to color in this figure legend, the reader is referred to the web version of this article.)

Regarding aroma and taste, the sensory profiles assessed by radar charts (Fig. 2B-C) differed markedly among treatments. For aroma, the NS sample was dominated by the highest floral, fresh, and grassy notes, reflecting minimal thermal conversion of green leaf odorants. The CT sample exhibited the most intense chestnut and beany aromas and the lowest grassy note, indicating a more pronounced conversion of green leaf odorants during shaping. This may be associated with the stronger stirring and kneading action of CT, which could enhance heat and mass transfer and thereby promote the transformation of volatile metabolites (Chen et al., 2026). Notably, the LT sample showed relatively high beany aroma and moderate chestnut, while the PB sample displayed balanced intermediate levels of chestnut and beany. For taste, the CT sample displayed the highest umami and the lowest bitterness and astringency, along with high thickness. In contrast, the LT sample exhibited moderate bitterness and astringency but the highest sweetness aftertaste. The PB sample presented a balanced profile with moderate bitterness, astringency, and moderate thickness, while the NS sample showed relatively high umami but a thin mouthfeel and low sweetness aftertaste, consistent with minimal cell disruption and limited release of polysaccharides and sugars (Wei et al., 2025). These differences in taste and aroma among the tested treatments underscore the critical role of the shaping process in modulating the overall flavor quality of Xinyang Maojian green tea.

### 3.2 Differentiation of green tea flavor profiles by electronic nose and electronic tongue

To objectively validate the aroma and taste differences observed in sensory evaluation, electronic nose (*E*-nose) and electronic tongue (E-tongue) analyses were performed. The E-nose PCA and radar plots (Fig. 3A-B) demonstrated clear separation of the CT process from the other three treatments (NS, PB, LT), which clustered together. This distinct profile was mainly driven by the stronger responses of the CT sample across nearly all sensor classes, including an exceptionally high response for the aromatics/aldehydes sensor. Such a response pattern suggests a greater contribution of aromatic and aldehyde volatiles to the aroma profile of CT, which is consistent with previous reports on the sensitivity of this sensor class to aromatic compounds (Wang et al., 2021b). This *E*-nose-based differentiation was also consistent with the sensory evaluation, in which CT was rated as having the most intense chestnut and beany aromas. The clustering of the other three treatments suggests that while their sensory aroma profiles show subtle differences, their overall volatile composition is more similar compared to the distinct CT profile. The E-tongue, as a non-compound-specific technique based on a multivariate sensor array and model training, has limited capacity to specifically resolve umami-related differences in tea infusions. Accordingly, its results were interpreted as supportive and complementary evidence rather than definitive proof of umami differences. Nevertheless, this technique was still informative for overall taste patterns. The E-tongue PCA (Fig. 3D) showed a clearer separation among the four shaping methods. The radar plot (Fig. 3C) showed that all samples exhibited very low saltiness and sourness responses, suggesting negligible off-flavors and a normal taste background. Among the main taste attributes, CT had the highest umami response but relatively low sweetness, and the lowest bitterness and astringency responses, consistent with its sensory profile. These integrated taste patterns may partly reflect the intense mechanical and thermal forces of the CT process, which could promote more thorough conversion of precursor compounds: vigorous mechanical disruption may facilitate the release of free amino acids (contributing to umami), while simultaneously driving the consumption of soluble sugars via enzymatic oxidation and other reactions and the breakdown or transformation of bitter/astringent catechins into less harsh derivatives (Shi et al., 2019; Wang et al., 2022). In contrast, the PB process, which applies relatively gentle stirring and kneading to shape the leaves, may have been associated with greater retention of bitter and astringent compounds, possibly contributing to the highest bitterness and astringency responses despite its moderate overall sensory scores. The Li Tiao treatment, being the most gentle manipulation, may best preserve soluble sugars, consistent with its highest sweetness response and a sweeter aftertaste. Pearson correlation analysis provided quantitative support for these instrumental-sensory associations (Fig. S2). *E*-nose sensors of ketones/aromatics, nitrogen compounds, and ketones/alcohols were significantly negatively correlated with grassy and floral notes (*r* = −0.86 to −0.84, *p* < 0.01) and positively correlated with chestnut and beany aromas (*r* = 0.79–0.82, *p* < 0.05). For taste, E-tongue bitterness and astringency sensors were highly correlated with the corresponding sensory scores (*r* = 0.993, *p* < 0.001), and the sweetness sensor was strongly correlated with the sweetness aftertaste (*r* = 0.972, *p* < 0.001). Notably, the E-tongue umami sensor was not significantly correlated with the sensory umami score (*r* = 0.356, *p* = 0.386), consistent with the limited compound-specific resolution of the E-tongue for umami noted above.

**Fig. 3:**
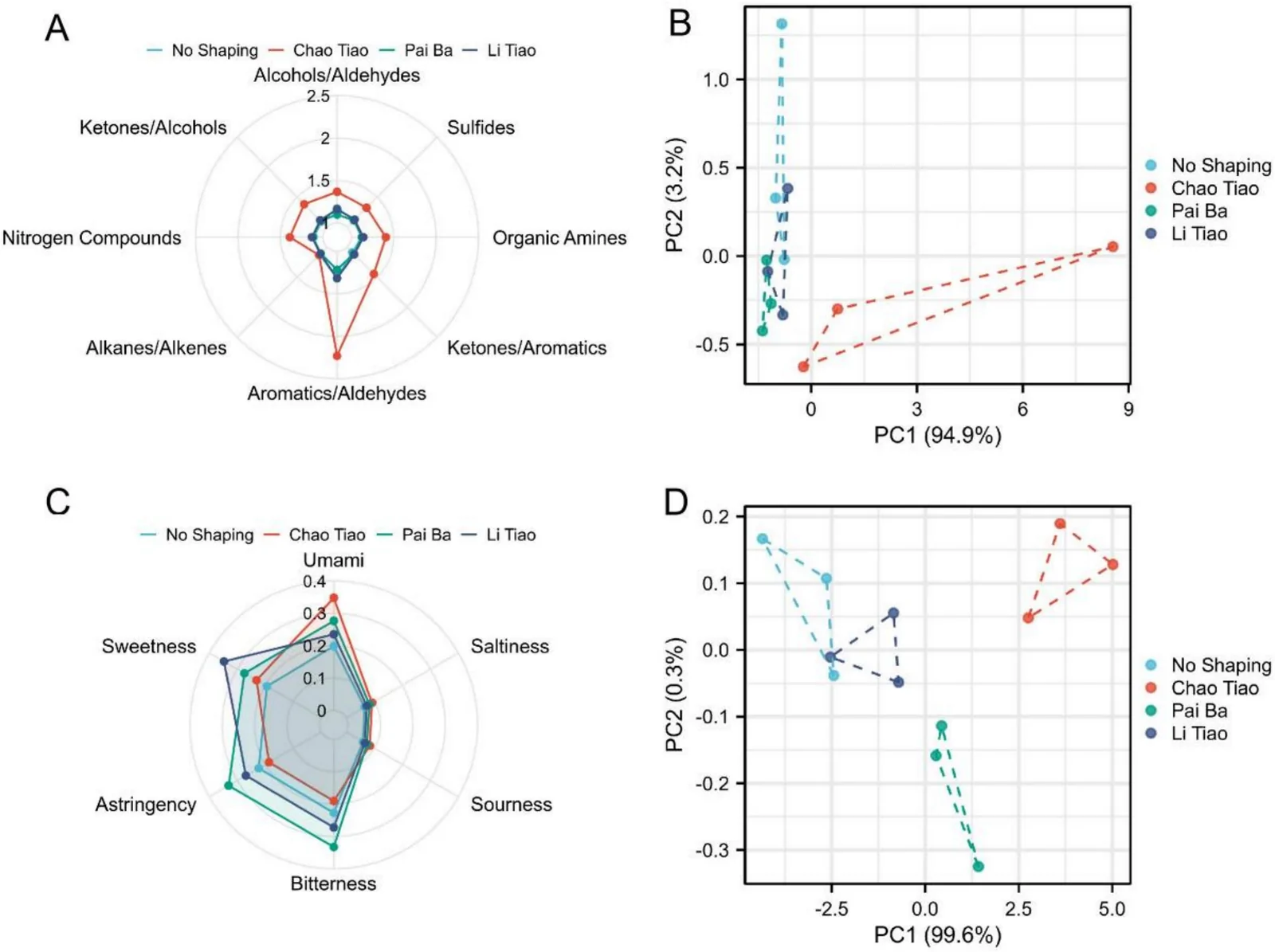
Combined electronic nose (*E*-nose) and electronic tongue (E-tongue) analysis of green tea processed by different shaping methods. (A) E-nose radar plot of sensor responses. (B) E-nose PCA score plot. (C) E-tongue radar plot of taste attributes. (D) E-tongue PCA score plot. (For interpretation of the references to color in this figure legend, the reader is referred to the web version of this article.)

### 3.3 Alterations in physicochemical composition induced by different shaping processes

As shown in Fig. 4A, the water extract content of all shaping treatments (CT, PB, LT) was higher than that of NS, with CT exhibiting the highest level followed by PB and LT. This is presumably because mechanical manipulation during shaping disrupts cell walls, enhancing the release of soluble solids, and the especially intense rolling and twisting in CT further amplifies this effect. Similar cell wall breakdown has been reported in tea processing (Feng et al., 2024), supporting the general contribution of cell-wall disruption to the release of soluble solids. Compared with NS, CT significantly increased the amino acid content, whereas LT and PB showed significantly lower levels. The mechanical stress experienced by LT and PB may promote oxidative or enzymatic degradation of free amino acids, while the vigorous disruption in CT likely facilitates protein hydrolysis but restricts subsequent degradation (Yu & Yang, 2020). For tea polyphenols, caffeine, and total catechins, all shaped samples showed higher contents than NS, with PB and LT exceeding CT. Ester-type catechins (EGCG, GCG, ECG) followed a similar pattern, with PB showing the highest EGCG and GCG and LT the highest ECG. The sum of ester-type catechins increased from 10.89% in NS to 12.54% in CT, an elevation of about 15.2% (Table 1). The non-ester catechins (EGC, EC, C) behaved differently, as EGC and EC were elevated in most shaped samples, except that EC in LT did not differ significantly from NS, and C remained unchanged. These results suggest that shaping facilitates the extraction of phenolic compounds and caffeine, but the more intense rolling in CT may promote oxidation or transformation, partially offsetting the extraction effect and leading to lower levels of polyphenols, caffeine, and some catechins in CT than in PB and LT (Kong et al., 2026). For soluble sugars (Fig. 4E), CT significantly reduced the content relative to NS, whereas LT and PB showed slightly higher levels (not significant). This difference reflects the contrasting mechanical intensities discussed above. The vigorous kneading of CT may accelerate sugar consumption, while the gentle, low-shear straightening of LT would cause only limited cell disruption, thereby preserving sugars. The same reasoning also accounts for the higher soluble sugars but lower free amino acids in LT than in CT. The flavonoids (Fig. 4F) were most abundant in NS, followed by PB and CT, with LT showing the lowest level. The high abundance in NS can be explained by the lack of thermal treatment, as heating during shaping would promote flavonoid degradation. Among the shaped samples, the lowest level in LT likely reflects its weakest mechanical action, which limited flavonoid release, whereas PB and CT, with stronger mechanical action, showed higher contents. These compositional shifts provide a robust chemical explanation for the instrument and sensory data. CT favors umami and mouthfeel due to its highest water extract and amino acids. Despite high catechins, the high amino acids likely mask bitterness and astringency via taste interactions, explaining the lowest bitterness and astringency. PB and LT, with lower amino acids, lack this masking. Their high polyphenols and catechins lead to pronounced bitterness and astringency. NS, with lowest catechins and moderate amino acids, yields a light clean taste. The high soluble sugars in LT account for its sweet aftertaste.

**Fig. 4:**
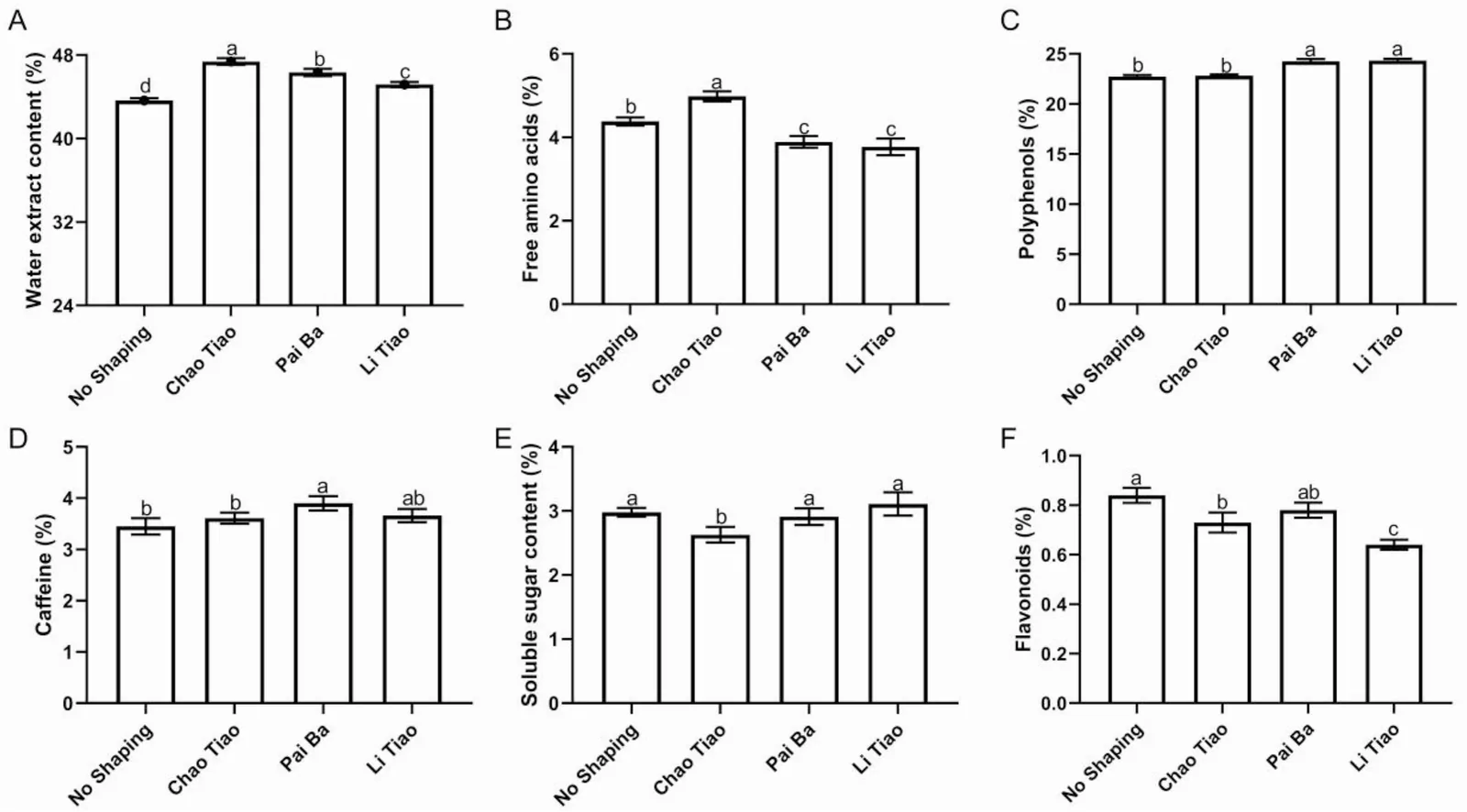
Effects of different shaping processes on the physicochemical properties of tea samples. (A) Water extract content. (B) Free amino acids content. (C) Polyphenols content. (D) Caffeine content. (E) Soluble sugar content. (F) Flavonoids content. Data are presented as mean ± standard deviation. Bars labeled with different lowercase letters indicate significant differences among treatments (*p* < 0.05).

**Table 1:** Catechin composition (%) of tea samples subjected to different shaping processes.

Treatments	EGC	C	EC	EGCG	GCG	ECG	Total catechins
No Shaping	1.50 ± 0.08b	0.09 ± 0.01a	0.63 ± 0.07b	8.76 ± 0.61c	0.12 ± 0.01c	2.01 ± 0.19c	13.11 ± 0.74c
Chao Tiao	1.70 ± 0.08a	0.11 ± 0.01a	0.78 ± 0.02a	10.01 ± 0.4b	0.17 ± 0.01b	2.36 ± 0.1b	15.13 ± 0.65b
Pai Ba	1.73 ± 0.13a	0.10 ± 0.01a	0.75 ± 0.04a	11.35 ± 0.47a	0.21 ± 0.02a	2.53 ± 0.19ab	16.67 ± 0.85a
Li Tiao	1.66 ± 0.05a	0.10 ± 0.02a	0.67 ± 0.03b	10.91 ± 0.85ab	0.16 ± 0.02b	2.61 ± 0.07a	16.11 ± 0.61ab

Note: Values are expressed on a dry weight basis. Different letters in the same column indicate significant differences (*p* < 0.05) among treatments.

### 3.4 Effects of different shaping processes on volatile metabolite profiles in Xinyang Maojian green tea

#### 3.4.1 Overall volatile metabolite profiles

The shaping process significantly influenced the volatile metabolite profiles of Xinyang Maojian green tea. The volatile metabolites were categorized into 14 classes (Fig. S3A), with terpenoids constituting the most abundant, followed by hydrocarbons, esters, and ketones, while sulfur and halogenated hydrocarbons were the least abundant. This distribution is consistent with previous reports where terpenoids are major contributors to green tea aroma due to their low odor thresholds and high volatility (Wei et al., 2023).

Regarding the shaping effects, CT exhibited the highest average total volatile content, with the highest relative contributions of amines, sulfur compounds, and heterocyclic compounds. PB showed a comparatively higher phenol content (Fig. S3B). LT was characterized by the highest ester content and relatively high alcohol and acid contents, while its heterocyclic contribution was the lowest. These differences may be related mainly to the intensity and mode of mechanical action: the more intense stirring and kneading during CT may promote the release and generation of amine- and sulfur-containing volatiles and heterocyclics, whereas the milder stirring and kneading in PB may better preserve phenolic precursors, and the relatively gentle manipulation in LT may favor the retention of alcohols, esters, and acids while limiting heterocyclic formation.

In terms of proportions, terpenoids consistently accounted for the largest fraction across all processes (24.20%–28.65%), while esters and ketones each exceeded 10% (Fig. S3C). Notable variation in the proportions of alcohols, phenols, and amines was also evident, further highlighting the critical role of shaping in modulating the volatile composition and, consequently, the sensory attributes of the final product.

#### 3.4.2 Analysis of key volatile metabolites

To identify the specific compounds driving the aroma differences observed above, a partial least squares-discriminant analysis (PLS-DA) was conducted. The scores plot (Fig. 5A) showed that quality control (QC) samples fell within an acceptable range, validating the reliability of the analysis. The model yielded R^2^X = 0.590, R^2^Y = 0.403, and Q^2^ = 0.189. To exclude the possibility of overfitting, a permutation test (*n* = 200) was performed, producing a negative Q^2^ intercept of −0.3 and permutation *p*-values <0.01 for both R^2^Y and Q^2^ (Fig. S4). Clear clustering of samples in the PLS-DA space confirmed that the shaping process significantly influences the volatile metabolite composition. Notably, CT samples were distinctly separated from the NS control, underscoring the pronounced impact of this process.

**Fig. 5:**
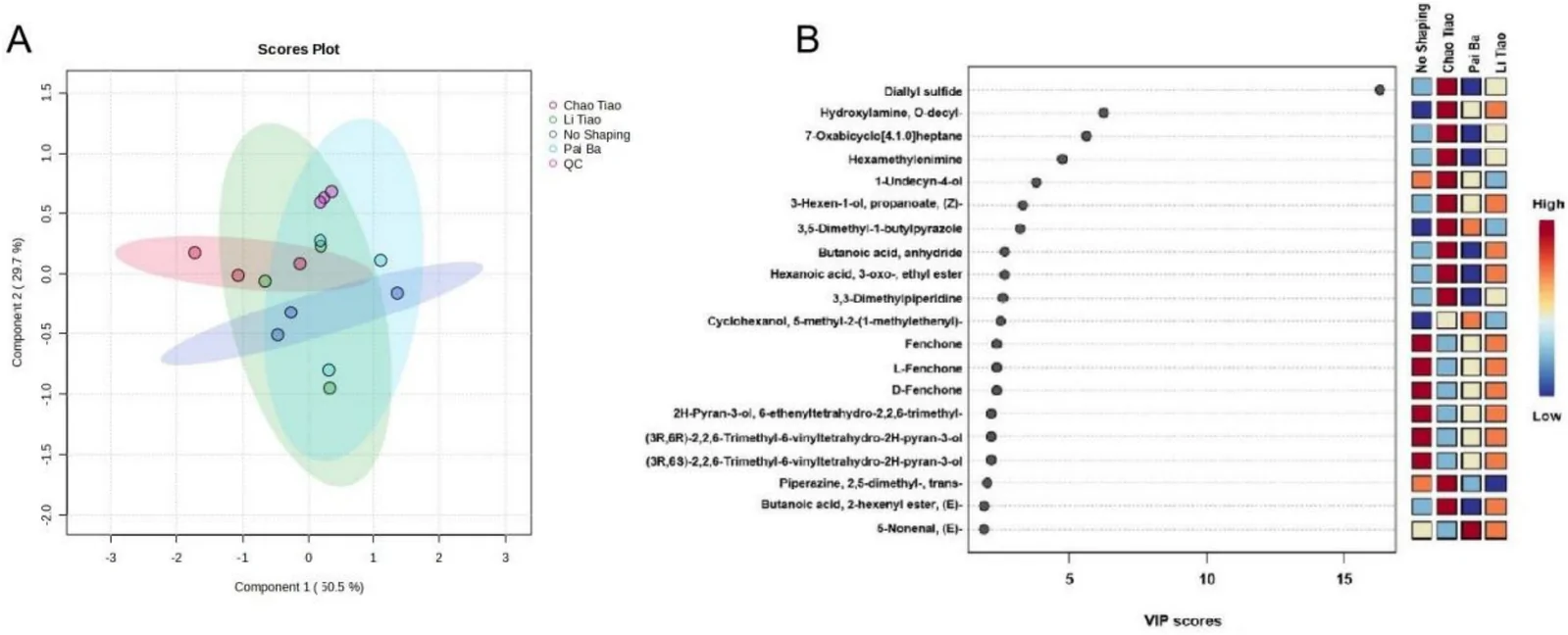
PLS-DA Analysis of Volatile Metabolites in Xinyang Maojian Green Tea under Different Shaping Processes. (A) PLS-DA Scores Plot. (B) Variable Importance in Projection (VIP) Scores Plot highlighting the top 20 volatile metabolites. Color intensity represents the relative increase (red) or decrease (blue) in metabolite content compared to the control. (For interpretation of the references to color in this figure legend, the reader is referred to the web version of this article.)

A total of 50 volatile metabolites with VIP scores >1 were identified (Table 2), predominantly classified as terpenoids (12), ethers (14), and heterocyclic compounds (7). Comparative analysis revealed that PB and CT generally led to a reduction in most terpenoid substances, whereas LT had minimal effect on them. Ether compounds decreased under PB but increased under LT. Heterocyclic compounds were enhanced under CT and LT, but suppressed under PB.

**Table 2:** Identification of volatile metabolites in different samples through GC–MS (VIP > 1).

Compounds	Formula	CAS	VIP	Relative content			
				No Shaping	Chao Tiao	Pai Ba	Li Tiao
Terpenoids (12)							
Furan, 3-(4-methyl-3-pentenyl)-	C10H14O	539–52-6	1.17	0.30	0.29	0.29	0.29
Fenchone	C10H16O	1195-79-5	2.35	0.51	0.49	0.49	0.51
p-Mentha-1,5-dien-8-ol	C10H16O	1686-20-0	1.14	0.37	0.31	0.29	0.30
Naphthalene,1,2,3,4-tetrahydro-1,6-dimethyl-4-(1-methylethyl)-, (1S-cis)-	C15H22	483–77-2	1.34	0.47	0.40	0.41	0.46
3-Oxatricyclo[4.1.1.0(2,4)]octane, 2,7,7-trimethyl-	C10H16O	1686-14-2	1.13	0.49	0.52	0.51	0.46
2,6-Dimethyl-2-trans-6-octadiene	C10H18	2609-23-6	1.25	0.25	0.23	0.25	0.24
Cyclohexanol, 5-methyl-2-(1-methylethenyl)-	C10H18O	7786-67-6	2.49	0.09	0.25	0.28	0.14
L-Fenchone	C10H16O	7787-20-4	2.35	0.51	0.49	0.49	0.51
D-Fenchone	C10H16O	4695-62-9	2.35	0.51	0.49	0.49	0.51
trans-Calamenene	C15H22	73,209–42-4	1.34	0.47	0.40	0.41	0.46
cis-Calamenene	C15H22	72,937–55-4	1.34	0.47	0.40	0.41	0.46
2,6-Octadiene, 2,6-dimethyl-	C10H18	2792-39-4	1.25	0.25	0.23	0.25	0.24

Sulfur compounds (1)							
Diallyl sulfide	C6H10S	592–88-1	16.32	1.76	2.92	1.48	1.91

Ketone (4)							
2(1H)-Pyridinone	C5H5NO	142–08-5	1.57	0.40	0.40	0.38	0.39
3,4-Dimethyl-1,2-cyclopentadione	C7H10O2	13,494–06-9	1.36	0.46	0.45	0.43	0.44
2-Cyclopenten-1-one, 3-ethyl-2-hydroxy-	C7H10O2	21,835–01-8	1.36	0.46	0.45	0.43	0.44
2-Hydroxy-3,5-dimethylcyclopent-2-en-1-one	C7H10O2	21,834–98-0	1.36	0.46	0.45	0.43	0.44

Hydrocarbons (1)							
cis-2,6-Dimethyl-2,6-octadiene	C10H18	2492-22-0	1.25	0.25	0.23	0.25	0.24

Heterocyclic compound (7)							
3,3-Dimethylpiperidine	C7H15N	1193-12-0	2.58	0.39	0.61	0.31	0.42
7-Oxabicyclo[4.1.0]heptane	C6H10O	286–20-4	5.63	0.67	1.13	0.54	0.80
Piperazine, 2,5-dimethyl-, trans-	C6H14N2	2815-34-1	2.00	0.23	0.38	0.17	0.11
Furan, 2-butyltetrahydro-	C8H16O	1004-29-1	1.23	0.10	0.17	0.12	0.13
3,5-Dimethyl-1-butylpyrazole	C9H16N2	2655-37-0	3.21	0.75	1.12	0.94	0.87
(3*R*,6*R*)-2,2,6-Trimethyl-6-vinyltetrahydro-2H-pyran-3-ol	C10H18O2	14,009–71-3	2.15	0.66	0.62	0.65	0.65
(3*R*,6*S*)-2,2,6-Trimethyl-6-vinyltetrahydro-2H-pyran-3-ol	C10H18O2	39,028–58-5	2.15	0.66	0.62	0.65	0.65

Ether (14)							
Benzene, 1-ethyl-4-methoxy-	C9H12O	1515-95-3	1.63	0.34	0.31	0.32	0.34
1-Octen-3-yl-acetate	C10H18O2	2442-10-6	1.65	0.43	0.44	0.43	0.43
3-Hexen-1-ol, propanoate, (*Z*)-	C9H16O2	33,467–74-2	3.31	0.92	1.00	0.92	0.94
3-Methylheptyl acetate	C10H20O2	72,218–58-7	1.28	0.13	0.07	0.04	0.12
2-Butoxyethyl acetate	C8H16O3	112–07-2	1.64	0.34	0.40	0.33	0.41
Butanoic acid, 2-hexenyl ester, (*E*)-	C10H18O2	53,398–83-7	1.90	0.24	0.38	0.24	0.35
2(5H)-Furanone, 3-hydroxy-4,5-dimethyl-	C6H8O3	28,664–35-9	1.77	0.72	0.72	0.72	0.67
Acetic acid, decyl ester	C12H24O2	112–17-4	1.23	0.30	0.34	0.26	0.39
Butanoic acid, anhydride	C8H14O3	106–31-0	2.64	0.61	0.75	0.60	0.70
n-Amyl isovalerate	C10H20O2	25,415–62-7	1.28	0.13	0.07	0.04	0.12
2-Hexen-1-ol, propanoate, (E)-	C9H16O2	53,398–80-4	1.51	0.25	0.39	0.21	0.37
Butanoic acid, 3-methyl-, 2-methylbutyl ester	C10H20O2	2445-77-4	1.28	0.13	0.07	0.04	0.12
Pentanoic acid, 3-methylbutyl ester	C10H20O2	2050-09-1	1.28	0.13	0.07	0.04	0.12
Hexanoic acid, 3-oxo-, ethyl ester	C8H14O3	3249-68-1	2.64	0.61	0.75	0.60	0.70

Amine (3)							
Benzeneethanamine	C8H11N	64–04-0	1.22	0.30	0.28	0.28	0.29
Hydroxylamine, O-decyl-	C10H23NO	29,812–79-1	6.25	1.11	1.42	1.13	1.33
Hexamethylenimine	C6H13N	111–49-9	4.74	0.55	0.94	0.46	0.65

Aldehyde (3)							
Non-8-enal	C9H16O	39,770–04-2	1.34	0.27	0.20	0.21	0.23
3-p-Menthen-7-al	C10H16O	27,841–22-1	1.54	0.14	0.12	0.09	0.14
5-Nonenal, (E)-	C9H16O	2277-18-1	1.89	0.92	0.84	1.07	0.98

Alcohol (4)							
2H-Pyran-3-ol, 6-ethenyltetrahydro-2,2,6-trimethyl-	C10H18O2	14,049–11-7	2.15	0.66	0.62	0.65	0.65
trans,cis-2,6-Nonadien-1-ol	C9H16O	28,069–72-9	1.31	0.23	0.07	0.07	0.21
1-Undecyn-4-ol	C11H20O	22,127–86-2	3.8	1.08	1.10	1.07	1.06
1-Octanol, 3,7-dimethyl-	C10H22O	106–21-8	1.23	0.13	0.25	0.14	0.22

Acid (1)							
4-Aminobutanoic acid	C4H9NO2	56–12-2	1.26	0.15	0.24	0.15	0.21

Note: Red and blue markings indicate respectively an increase and decrease in relative content under different treatments compared to the control (*p* < 0.05).

We focused on the top 20 VIP metabolites (Fig. 5B). Several compounds including diallyl sulfide, hydroxylamine o-decyl-, hexamethylenimine, 3-hexen-1-ol propanoate, 3,5-dimethyl-1-butylpyrazole, and (*E*)-butanoic acid 2-hexenyl ester were significantly higher in the CT process compared to others. Notably, these compounds are predominantly heterocyclic nitrogenous (e.g., 3,5-dimethyl-1-butylpyrazole) or sulfur-containing volatiles (e.g., diallyl sulfide). Their enrichment suggests that the intense mechanical rolling during CT promoted complex degradation reactions. Such nitrogen- and sulfur-heterocycles are known to impart nutty and roasty notes in roasted foods (Zhang et al., 2026), which is consistent with the intense chestnut and beany aromas recorded in sensory evaluation. The simultaneous elevation of multiple heteroatom-bearing volatiles in CT also provides a plausible chemical basis for its exceptionally high response in the aromatics/aldehydes sensor of the *E*-nose, which is sensitive to a broad range of polar and aromatic volatiles, including those with nitrogen and sulfur functional groups. Conversely, fenchone and its isomers—belonging to the monoterpenoid ketone class—as well as certain pyran derivatives, were reduced across all three shaped processes relative to NS. The reduction of these terpenoids, especially in CT, likely reflects their mechanical degradation. The loss of such volatiles, which typically contribute floral, herbaceous, or cooling notes, enhances the perceptual prominence of roasting-derived compounds by reducing their sensory masking effect (Ravichandran & Parthiban, 1998).

### 3.5 Effects of different shaping processes on non-volatile metabolite profiles in Xinyang Maojian green tea

#### 3.5.1 Overall non-volatile metabolite profiles

The non-volatile metabolome was comprehensively profiled, leading to the annotation of 5406 metabolites, which were consolidated into six major categories (Fig. S5A). To assess the global impact of shaping processes, the relative proportion of each major category was calculated (Fig. S5B). Remarkably, the overall compositional profile remained relatively stable across all treatments. Nitrogenous compounds and phenolic compounds consistently constituted the dominant fractions, together accounting for approximately two-thirds of the total relative abundance (∼34% and ∼ 32%, respectively), regardless of the shaping method. This stability suggests that the basic architecture of the non-volatile metabolic pool was largely conserved under different mechanical manipulations. We therefore postulated that the effect of shaping might not reside in altering the gross quantity of major categories, but rather in fine-tuning the abundance of specific, key metabolites within these stable pools. This hypothesis directed our subsequent analysis towards identifying differential metabolites at a more refined level, in order to explain the distinct sensory profiles observed earlier.

#### 3.5.2 Analysis of key non-volatile metabolites modulated by shaping processes

Multivariate analysis clearly distinguished the metabolic profiles of tea leaves subjected to different shaping processes (Fig. S6A). The supervised PLS-DA model revealed distinct clustering among the four treatment groups, with the first two principal components explaining 15.3% and 13.2% of the variance, respectively. The model parameters (R^2^X = 0.407, R^2^Y = 0.648, Q^2^ = 0.261) combined with permutation testing (*n* = 200, Q^2^ intercept = −0.4, permutation *p*-values <0.01 for R^2^Y and Q^2^, Fig. S6B) demonstrated the robustness of the model and confirmed that the observed separation was not due to overfitting. This pronounced separation indicates that each shaping process imposed a significant and unique reprogramming of the leaf metabolome, providing a robust basis for identifying key discriminatory metabolites that underlie the distinct sensory and taste characteristics of the resulting teas. Targeted analysis of the 88 significantly altered metabolites (VIP > 1.5, *p* < 0.05) revealed that the shaping processes fine-tuned specific pathways within major chemical classes (Fig. 6). Detailed results for each class are presented below.

**Fig. 6:**
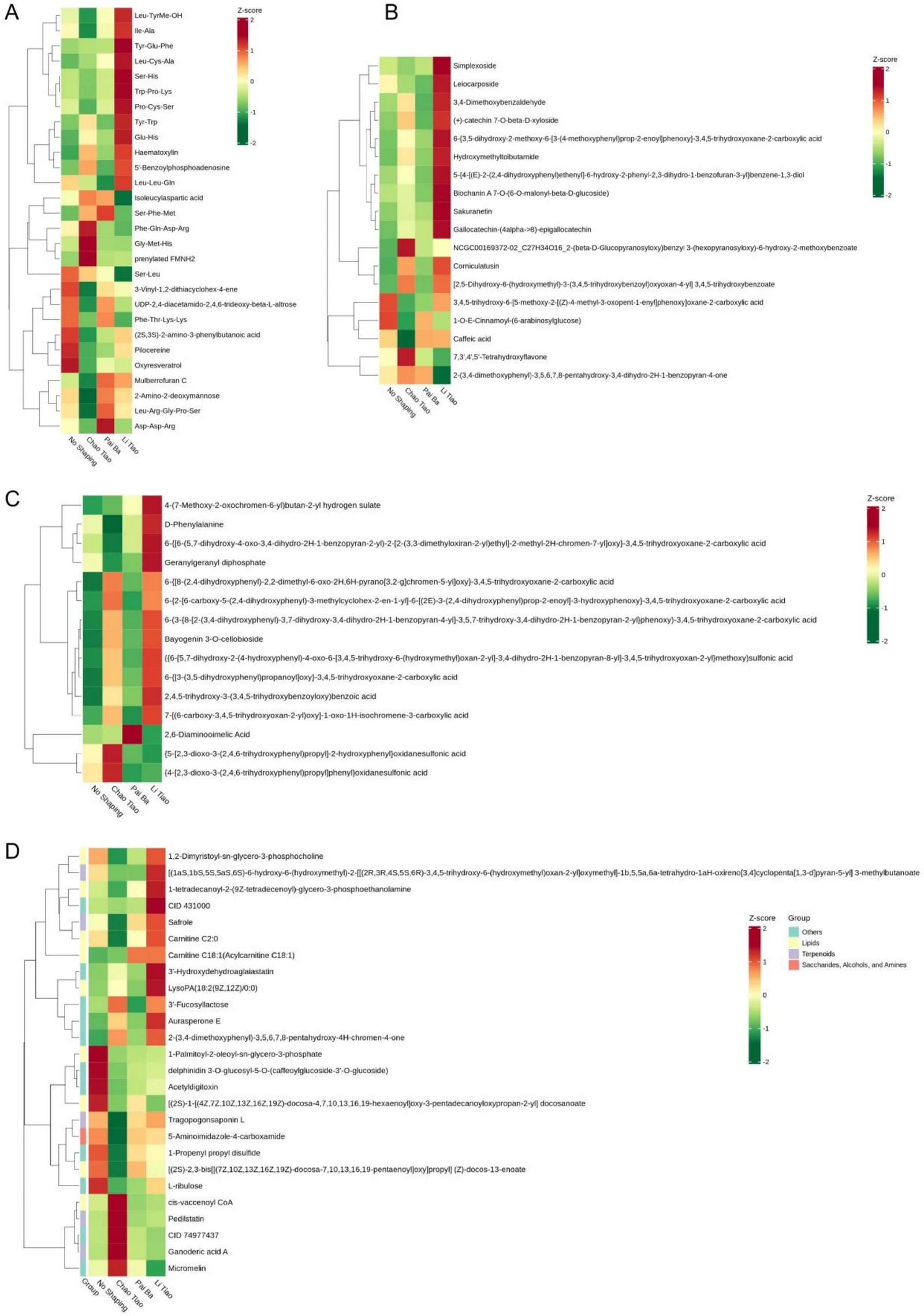
Heatmap analysis of differential non-volatile metabolites grouped by chemical class. Hierarchical clustering heatmaps display the row-wise *Z*-scores of the mean relative abundance for differential metabolites (VIP > 1.5, *p* < 0.05) across the four shaping processes. Metabolites are grouped into four panels: (A) Nitrogenous compounds, (B) Phenolic compounds, (C) Organic Acids and (D) Lipids, Terpenoids and others.

The shaping processes induced distinct and compound-specific alterations in the nitrogenous metabolite pool. LT broadly elevated most di- and tripeptides, with Glu-His (a known umami-enhancing dipeptide) and Tyr-Trp (a potential kokumi-active peptide) reaching their highest levels. The sustained mechanical vibration during LT may promote protein degradation and peptide release without causing the severe tissue disruption associated with stronger shear forces (Kumar et al., 2023). In contrast, CT exhibited a more selective regulatory profile: Gly-Met-His, Phe-Gln-Asp-Arg, and prenylated FMNH₂ were dramatically elevated up to 3–4.6 times higher than NS, while Ile-Ala, Phe-Thr-Lys-Lys, and oxyresveratrol were markedly reduced. The opposing trends in peptide abundance may reflect a dynamic balance between generation and degradation: the rotary kneading and compression during CT may preferentially liberate certain peptides (e.g., Gly-Met-His, Phe-Gln-Asp-Arg) from storage proteins, while concurrently accelerating the degradation of less stable peptides (e.g., Ile-Ala, Phe-Thr-Lys-Lys), leading to their depletion. Notably, the elevation of Gly-Met-His and Phe-Gln-Asp-Arg (containing sulfur or charged residues) may contribute to complex umami and mouthfeel, while the depletion of many other peptides could explain why CT tea maintains a balanced, clean taste (Ramesh et al., 2025). However, PB showed only limited changes relative to NS, which may reflect the complexity of mechanical processing, possibly involving synergistic interactions among multiple parameters and threshold-dependent biological responses.

Regarding phenolic metabolites, LT broadly elevated the majority of compounds in this class, including flavonoid glycosides (e.g., biochanin A 7-O-(6-O-malonyl-β-D-glucoside), gallocatechin-(4α → 8)-epigallocatechin, (+)-catechin 7-O-β-D-xyloside), methylated flavanones (e.g., Sakuranetin), and benzaldehyde derivatives (e.g., 3,4-dimethoxybenzaldehyde). This broad increase may be attributed to the mild but continuous reciprocating agitation during LT, which could promote the release of cell-wall-bound phenolics and stimulate phenylpropanoid metabolism without causing severe tissue disruption, thereby preserving or enhancing these compounds (Das et al., 2020). CT exhibited a highly selective regulation of phenolic metabolites. The most striking feature was the drastic reduction of caffeic acid, a key astringency-related phenolic acid (Shen et al., 2025). This reduction likely reflects its degradation or polymerization under the intense mechanical stirring of CT, potentially lowering harsh astringency. Meanwhile, (+)-catechin 7-O-β-D-xyloside and corniculatusin were significantly elevated, and several unique glycosylated phenolic acids and ellagitannins were dramatically increased, while 1-O-*E*-cinnamoyl-(6-arabinosylglucose) and a related compound were markedly reduced. The increase in glycosylated catechins may contribute to a more lingering, complex mouthfeel, as glycosylation often reduces bitterness/astringency while enhancing solubility and interaction with salivary proteins (Xie et al., 2026). PB showed only limited changes in phenolic metabolites relative to the control, possibly because its mechanical action, mainly rotational kneading at moderate intensity, neither caused the pronounced cell disruption associated with CT nor provided the sustained mechanical stimulus that may underlie the broad metabolic response in LT.

Compared with NS, all three shaping treatments generally upregulated the levels of most organic acid metabolites, indicating that thermal and mechanical effects associated with shaping may be a common factor contributing to the accumulation of these compounds. Under LT, glycosylated and sulfated organic acid derivatives (e.g., bayogenin 3-O-cellobioside) were broadly elevated. This may be related to the reciprocating vibration used in LT to straighten the leaves into strips, which involves repeated friction and mild compression of the leaf tissues and may affect the release or retention of bound organic acid derivatives. Notably, geranylgeranyl diphosphate (GGPP), a key terpenoid precursor, was approximately 2.1-fold higher in LT than in the other treatments, suggesting that the relatively mild mechanical action of LT may be associated with greater retention of this heat-labile diphosphate intermediate, possibly through reduced degradation or downstream conversion. This may be consistent with the fresh, clean aroma profile of LT-processed tea. In addition, a few compounds (e.g., two sulfated phenylpropanoids) were lower in LT. This decrease was limited to a small number of compounds and may reflect the distinct processing conditions of LT, which could shift the balance between sulfated conjugates and their free or otherwise conjugated forms. Under CT, significant enrichment was observed for complex glycoside derivatives bearing caffeoyl and galloyl groups, together with the two sulfated phenylpropanoids. These compounds are expected to contribute to the astringency and antioxidant capacity of the tea infusion (Ke et al., 2025). Meanwhile, GGPP decreased in both CT and PB. This shared consumption pattern may be associated with the relatively strong mechanical action of CT and PB, which may promote hydrolysis of the diphosphate group or downstream conversion of GGPP. As a key precursor of terpene aroma compounds (e.g., linalool, geraniol), GGPP consumption suggests earlier or more extensive terpene formation in CT and PB. Notably, the GGPP decrease was more pronounced in CT than in PB. This may be consistent with the intense “chestnut/roasty” aroma typically associated with the prolonged pan-firing step during CT shaping, which may promote the conversion of terpenoid precursors into volatile aroma compounds.

The three shaping treatments induced distinct changes within the lipids, terpenoids, and other miscellaneous metabolites. Several polyketide and flavonoid derivatives (e.g., Aurasperone E, 2-(3,4-dimethoxyphenyl)-…) were markedly elevated in both CT and LT. These compounds serve as chromogenic precursors: flavonoids form the yellow infusion color, while polyketides generate brown pigments via oxidation (Patil & Murumkar, 2024), thereby likely enhancing the yellowness of the liquor and deepening the dry leaf color. The triterpenoid ganoderic acid A and the lignan maglifloenone were strongly enriched only in CT, and both possess antioxidant and free radical scavenging activities (Wu et al., 2024), suggesting that CT processing may enhance the functional quality of the final tea product. While the levels of saponin tragopogonsaponin L, carnitine derivative carnitine C2:0, several phospholipids (e.g.1,2-dimyristoyl-sn-glycero-3-phosphocholine), and the alkaloid 5-aminoimidazole-4-carboxamide were decreased. The former three may undergo hydrolysis or related degradation, yielding volatile aldehydes and ketones that enhance aroma complexity, whereas the latter may reflect altered purine metabolism that could affect umami nucleotide profiles (Andres-Hernando et al., 2021).

Collectively, these results suggest that shaping processes influence tea quality through distinct metabolic reprogramming. CT was characterized by selective enrichment of antioxidant triterpenoids/lignans and umami/kokumi peptides, together with depletion of astringent phenolics (e.g., caffeic acid) and potential conversion of lipid/saponin precursors into volatile aroma compounds, which may align with its intense chestnut/roasty aroma and balanced, umami-rich taste. PB exhibited fewer changes, consistent with its less intense mechanical action than CT. LT broadly accumulated small peptides, phenolic glycosides, and organic acids, consistent with its relatively mild mechanical action, which may support its fuller, richer mouthfeel and fresh aroma.

## 4 Conclusions

Collectively, this study suggests that shaping processes are important factors influencing metabolic reprogramming in Xinyang Maojian green tea and are associated with distinct quality outcomes. The Chao Tiao (CT) process showed the most pronounced changes in both volatile and nonvolatile metabolomes. In volatiles, the relatively strong mechanical action in CT may have promoted the formation of heterocyclic compounds and the depletion of monoterpenoid ketones, possibly contributing to the intense chestnut/beany aroma and the higher electronic nose response. In nonvolatiles, CT showed selective decreases in astringent phenolics (e.g., caffeic acid), increases in specific umami and kokumi peptides, and a decrease in geranylgeranyl diphosphate (GGPP). These changes may contribute to a more balanced umami taste with reduced bitterness and astringency, in line with its highest water extract and amino acid contents among the shaping treatments. In contrast, Li Tiao (LT) broadly accumulated small peptides and phenolic glycosides, possibly related to its relatively mild mechanical action, which may support a fuller mouthfeel and a sweet aftertaste, despite reduced free amino acids. Overall, these findings may help link shaping processes to flavor complexity and provide potential insights for the targeted production of high-quality green tea.

## CRediT authorship contribution statement

**Jinlei Luo:** Writing – original draft, Visualization, Investigation, Funding acquisition, Formal analysis, Data curation. **Yi Chen:** Writing – review & editing, Writing – original draft, Supervision, Project administration, Methodology, Formal analysis, Conceptualization. **Lingzhi Chen:** Validation, Investigation, Data curation. **Peng Yin:** Software, Resources, Investigation. **Guiyi Guo:** Writing – review & editing, Supervision, Resources, Methodology, Funding acquisition, Conceptualization. **Zhihui Wang:** Writing – review & editing, Supervision, Resources, Methodology, Conceptualization.

## Declaration of competing interest

The authors declare that they have no known competing financial interests or personal relationships that could have appeared to influence the work reported in this paper.

## Data Availability

Data will be made available on request.
