# Supplementary material for: Effects of different shaping processes on the quality, volatile and non-volatile metabolites of Xinyang Maojian green tea
Source: Food Chem X. 2026 Oct 28;40:104722. doi: 10.1016/j.fochx.2026.104722 (PMC13742501; doi:10.1016/j.fochx.2026.104722)
Supplement: Supplementary file 1 [file mmc1.docx]

**Supplementary Material**

**Effects of different shaping processes on the quality, volatile and non-volatile metabolites of Xinyang Maojian green tea**

**Jinlei Luo^a,1^, Yi Chen^a,1,*^, Lingzhi Chen^a^, Peng Yin^a^, Guiyi Guo^a,*^, Zhihui Wang^b,*^**

**^a^School of Tea Science, Xinyang Agriculture and Forestry University, Key Laboratory of Development and Utilization of Tea and Characteristic Plant Resources in South Henan, Xinyang, Henan, China**

**^b^Jiangxi Cash Crops Research Institute, Nanchang, Jiangxi, China**

*** Corresponding author.**

***E-mail addresses:* [chenyitea@163.com](mailto:chenyitea@163.com) (Y. Chen), [2505482155@qq.com](mailto:2505482155@qq.com) (G.-Y. Guo), [wzh1246900265@163.com](mailto:wzh1246900265@163.com) (Z.-H. Wang)**

**^1^ These authors contributed equally to this work.**

**Fig. S1.** PCA score plots of the volatile (A, B) and non-volatile (C, D) datasets generated using the current and alternative missing-value imputation strategies. A and C: current strategy (volatile: 1/5 of the minimum value; non-volatile: >50% missingness removal followed by KNN); B and D: alternative strategy (volatile: KNN; non-volatile: 1/5 of the minimum value). No substantial differences in the separation patterns were observed.

**Fig. S2.** Pearson correlation heatmaps between (A) E-nose sensor responses and aroma attributes and (B) E-tongue sensor responses and taste attributes. Colors represent Pearson correlation coefficients (r); asterisks indicate significance (*p < 0.05, **p < 0.01).

**Fig. S3.** Analysis of volatile metabolites in Xinyang Maojian green tea under different shaping processes. (A) Total number of volatile metabolites across various compound classes. (B) Relative content of volatile metabolites in different shaping processes. (C) Percentage composition of volatile metabolites under different shaping processes, highlighting the proportion of each compound class.

**Fig. S4.** Permutation test of the PLS-DA model for volatile metabolites (n = 200).

**Fig. S5.** Analysis of non-volatile metabolites in Xinyang Maojian green tea under different shaping processes. (A) Total number of annotated non-volatile metabolites across the six consolidated chemical classes. (B) Percentage composition of the six major non-volatile metabolite classes under different shaping processes, highlighting their relative proportions within the total metabolite pool.

**Fig. S6.** Multivariate analysis discriminates the metabolic profiles of different tea shaping processes. (A) Score plot from the partial least squares-discriminant analysis (PLS-DA). (B) Permutation test plot (n=200) for the PLS-DA model validation.
